# Early life disadvantage strengthens flight performance trade-offs in European starlings, *Sturnus vulgaris*

**DOI:** 10.1016/j.anbehav.2015.01.016

**Published:** 2015-04

**Authors:** Daniel O'Hagan, Clare P. Andrews, Thomas Bedford, Melissa Bateson, Daniel Nettle

**Affiliations:** Centre for Behaviour and Evolution & Institute of Neuroscience, Newcastle University, Newcastle, U.K.

**Keywords:** developmental stress, early life adversity, flight performance, locomotor performance, starlings

## Abstract

Developmental stress has been shown to affect adult flight performance in birds, with both negative and positive effects reported in the literature. Previous studies have used developmental manipulations that had substantial effects on patterns of growth. They have also examined mean levels of flight performance per individual, rather than investigating how developmental stress might alter trade-offs between different components of flight performance. We recorded multiple components of escape flight performance in 20 adult European starlings previously subjected to a manipulation likely to have altered levels of developmental stress. Siblings had been cross-fostered to nests where they were either slightly larger (advantaged treatment) or slightly smaller (disadvantaged treatment) than their competitors. The manipulation had no detectable effect on growth. However, developmental treatment affected performance in escape flights a year later by strengthening the trade-offs between different flight parameters. Disadvantaged birds faced a steeper trade-off between take-off speed and take-off angle, and a steeper trade-off between take-off angle and total time in flight, than advantaged birds. The results suggest that even subtle early life adversity that has no obvious effect on growth or size can leave a lasting legacy in the form of constraints on locomotor performance later in life.

## Highlights

• We studied flight performance in young adult starlings.
• The birds had either been advantaged or disadvantaged in the nest.
• Disadvantaged birds had to sacrifice more angle to sustain a given take-off speed.
• Even very subtle early life stressors can affect locomotion as an adult.

The abilities to take flight rapidly and manoeuvre accurately are vital to the survival of small birds (Cresswell, 1993). Variation in these abilities is determined by such factors as muscular power relative to mass, wing size and feather condition (Kullberg, Fransson, & Jakobsson, 1996; Swaddle, Witter, Cuthill, Budden, & McCowen, 1996; Verspoor, Love, Rowland, Chin, & Williams, 2007). Across many taxa, it has been shown that developmental stress can produce alterations in morphological and physiological parameters that persist into adulthood (e.g. Criscuolo, Monaghan, Nasir, & Metcalfe, 2008; Tschirren, Rutstein, Postma, Mariette, & Griffith, 2009; Verhulst, Holveck, & Riebel, 2006), and adult locomotor performance can be affected by conditions experienced early in life (Alvarez & Metcalfe, 2005; Álvarez & Metcalfe, 2007).

In birds, several studies have detected negative consequences of developmental stress for flight performance, generally finding these to become more marked with age (European starlings: Verspoor et al., 2007; zebra finches, *Taeniopygia guttata*: Criscuolo et al., 2011; mourning doves, *Zenaida macroura*: Miller, 2011). This is an example of a ‘silver spoon’ effect (Monaghan, 2008), whereby benign early conditions carry over into improved phenotypic status later in life. In contrast, two studies have shown that maternal stress or increased exposure to maternal stress hormones can lead to increased wing size and improved flight performance at maturity (great tits, *Parus major*: Coslovsky & Richner, 2011; European starlings: Chin et al., 2009). This would be an example of adaptive developmental plasticity, where an early stressor triggers an evolved anticipatory response to adverse situations later in life, and hence improved performance in some capacities. Both of the studies reporting positive effects used prehatching stressors (maternal predator exposure and embryonic corticosterone exposure), whereas all the studies reporting negative effects used posthatching stressors. Thus, pre- and post-hatching stress exposures may play different roles, with prehatching exposure more able to induce anticipatory adaptive plasticity, and posthatching stress more likely to impose constraints on adult phenotypic quality.

All of the developmental stress manipulations studied so far in the context of flight performance have affected growth in obvious ways, either altering the developmental trajectories of body and wing size, or prolonging the growth period. Thus it is possible that effects of developmental stress on flight performance are wholly due to consequent differences in overall size or catch-up growth. We recently developed a subtle early life manipulation in the European starling in which siblings were cross-fostered on day 2 of life to nests in which they were either slightly larger than the other chicks (the advantaged treatment) or slightly smaller (the disadvantaged treatment). The size differences were of the order of 5 g against an average day 2 weight of 13 g, and thus similar in magnitude to the difference between first- and last-hatching chicks in a natural nest. Previous studies using similar manipulations suggest that the disadvantaged birds would have had to beg more in order to be fed, and would have been jostled to more peripheral positions in the chick mass (Cotton, Wright, & Kacelnik, 2013). Our manipulation appeared to affect developmental stress, since telomere attrition, an acknowledged biomarker of developmental stress exposure, was greater in the disadvantaged than the advantaged group (Nettle et al., 2015). However, the manipulation had no detectable impact on the timing of weight gain, or weight or wing length at fledging. Thus, if there were any consequences of developmental treatment for adult flight performance in our birds, this would suggest that the impact of developmental stress on flight performance is not wholly due to variation in the pattern of overall growth.

Previous studies in this area have reduced flight performance to a single index, and often used only a single flight or the mean of several flights to represent each bird. Although convenient, this strategy masks the fact that there are several components to flight performance, and there may be trade-offs between them. Within the domain of take-off ability, birds face a trade-off between take-off speed and take-off angle, with one of these parameters being defended at the expense of the other when capacity is limited (Kullberg et al., 1996; Lind, Jakobsson, & Kullberg, 2010; Witter, Cuthill, & Bonser, 1994). Since developmental stress could influence muscular or metabolic capacity, it might affect the resolution or the severity of this trade-off. Once airborne, there may be a trade-off between flight speed and accuracy of a manoeuvre (Brilot, Asher, & Bateson, 2009). Again, developmental stress could affect the strength or resolution of the trade-off.

In this study, then, we measured key components of flight performance (take-off speed, take-off angle, time to reach a destination and accuracy of airborne manoeuvres) in European starlings from the cohort that had experienced the early life manipulation described above, recording several flights per bird wherever possible. This allowed us to examine impacts of early disadvantage on both average flight performance parameters and the trade-offs between different components of flight performance for each individual bird.

## Methods

### Subjects and Housing

Subjects were 26 European starlings (14 male:12 female, 14:12 from the two developmental treatments), taken from the wild as nestlings. When not in experimental procedures, birds were housed in groups of up to 20 in two large indoor aviaries (215 × 340 cm and 220 cm high; ca. 18 °C; 40% humidity; 15:9 h light:dark cycle), provided with environmental enrichment (foraging substrate, water baths, multilevel rope perches, cover) and clean drinking water, and fed ad libitum on domestic chick crumbs supplemented with cat biscuits (Royal Canin Ltd. ‘Fit’), dried insect food (Orlux insect patée), live mealworms, *Tenebrio molitor*, and fruit. The birds were maintained in nonbreeding condition at all times by the use of an unchanging light/dark cycle of long days.

### Developmental Manipulation

The developmental manipulation is described in full elsewhere (Nettle et al., 2015). Briefly, on posthatching day 2, quartets of siblings were removed from the natal nest and cross-fostered to two different host nests: the two in the advantaged (ADV) condition to a nest in which they were (mean + SD) 4.9 + 1.9 g larger than all other nestlings, and the two in the disadvantaged (DIS) condition to a nest in which they were 4.8 + 2.2 g smaller than all other nestlings. On posthatching day 12, they were removed to the laboratory where the natal families were reconstituted and chicks were hand-reared to independence, after which they lived in common aviaries. The manipulation led to no significant differences by treatment in body weight at any age (measured at days 3, 4, 7, 12, 15, 18, 21 and 24), although the ADV birds remained significantly heavier than their nest competitors at day 12, while the DIS birds remained significantly smaller than their nest competitors. Wing lengths did not differ significantly by treatment at day 12 or after fledging on day 24. The current experiment used a sample of the cohort, with individuals from eight natal families. The birds were 10–13 months old at the time of this experiment.

### Apparatus

**Figure 1:**
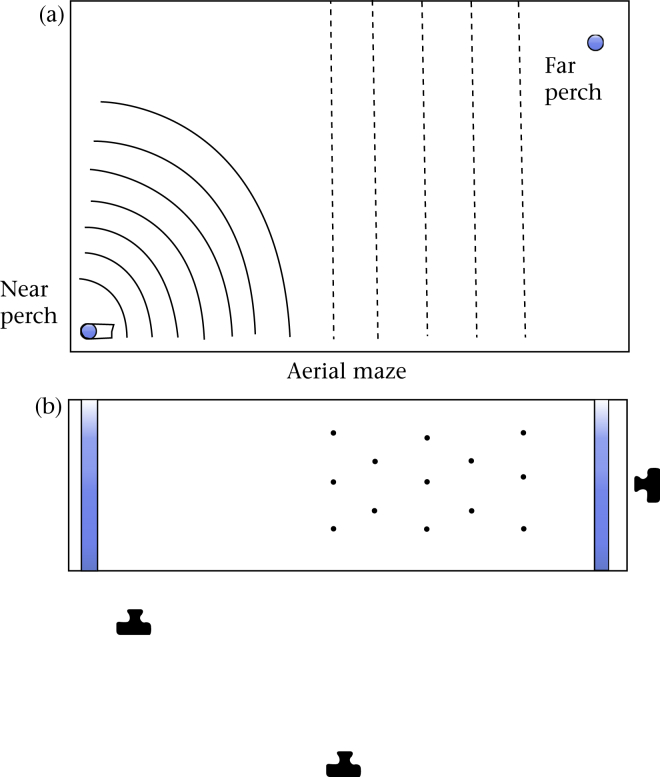
The experimental arena. (a) Side view showing perch locations, measuring arcs and aerial maze. (b) Plan view indicating positions of the three cameras.

We designed an experimental arena to measure both take-off performance and aerial manoeuvrability within the same trial. The arena was housed in an indoor room maintained at approximately 18 °C and 40% humidity on a 15:9 h light:dark cycle. No people or other birds were present in the room during the trials; the experimenter observed from outside the room using a video monitor. The arena (0.7 × 3 m and 2 m high; Fig. 1) was constructed from metal mesh, with two perches, one 10 cm from the floor at the near end, and the other 27 cm from the ceiling at the far end, the latter surrounded by cardboard for cover. During trials, a feeder containing live mealworms was placed close to the near perch. Also adjacent to the near perch was an electronic doorbell that produced the startle stimulus (the sound of a dog barking). The bird came to feed at the feeder and, during feeding, we triggered the doorbell, causing the bird to escape to the far, high perch. On the wall adjacent to the near perch were seven arcs marked in 15 cm increments from the perch. One metre from the near perch, the bird encountered an aerial maze consisting of 13 weighted strings, 25 cm apart, hanging from the ceiling in staggered rows of two or three. Three digital video cameras gave full coverage of the entire flight: one from the far perch (frame rate 30 fps), and two from the side of the arena, one covering its whole length (75 fps) and one covering the take-off area (50 fps).

### Procedure

On the afternoon of day 0, a bird was caught from the aviary in the dark, weighed using a digital balance (to the nearest 0.1 g), and measured using a wing rule (wing length to the nearest mm, average of two sides). Birds were assessed for feather damage such as broken primaries. Only four birds were judged to show any sign of damage; including damage in the analysis had no effect and damage is not considered further in this paper.

The bird was then released into the arena for a habituation phase starting at 1600 hours. The aerial maze was not lowered until 1700 hours and, during the habituation phase, the arena was enriched with extra intermediate perches, a water bath and food (chick crumbs and Orlux). To counteract the negative effects of isolation and encourage the bird to come to feed, a stuffed model conspecific was placed on the outside of the arena close to the feeder at the near perch. The flight trials began at approximately 0930 hours on day 1. The arena was cleared of enrichments, and the standard food in the feeder at the near perch was replaced with more highly valued mealworms. The experimenter waited until the bird came to feed, and allowed at least one beak dip into the food before triggering the startle stimulus.

After an escape flight, the startle stimulus was not triggered again for at least 5 min. After this interval, further escape flights were triggered when the bird came to feed. We were primarily interested in escape flights triggered by the startle stimulus, since these most closely represent the escape of a bird from a predator attack, and differ from routine flights (Veasey, Metcalfe, & Houston, 1998). However, if a bird had completed three escape flights, we allowed it to feed and captured a routine (nonstartled) flight from the perch. After this, if the bird continued to come down to feed, we captured up to two more escape flights. The sequence of escape and routine flights was not identical for every bird due to unplanned routine flights during the trials. At 1400 hours on day 1, the bird was removed from the arena if it had completed at least two escape flights (if it had completed one, the session was prolonged until 1500 hours). Otherwise, it returned to the habituation phase until 0930 on day 2, when the procedure was repeated. At the end of day 2, the bird was returned to the aviary regardless of how many flights had been completed.

### Measurement of Flight Performance

All flight performance measures were assessed from video blind to developmental treatment. For take-off speed and take-off angle, videos were paused 0.2 s after the bird's feet left the near perch. Using the software package ImageJ (Schneider, Rasband, & Eliceiri, 2012) and the known distances of the arcs, we calculated speed to that point (cm/s) and angle of ascent (degrees). We estimated total time of flight by counting the frames between the bird's feet leaving the near perch and arriving at the far perch, and using the camera frame rate to convert to milliseconds. Manoeuvrability was assessed by the number of strings hit. This was scored independently by two experimenters, and proved difficult to score accurately due to spontaneous movement of strings caused by airflow. There were discrepancies between the experimenters in 43% of cases; these were resolved by rescoring those videos together.

### Statistical Analysis

Data were analysed using linear mixed models (package ‘nlme’) in R (R Core Development Team, 2013), incorporating nested random effects for bird (where appropriate) and natal family to account for the structure within the data. We had a number of covariates potentially relevant to flight performance (see below), and were interested in whether developmental treatment explained additional variation above and beyond these covariates. We thus employed a model selection approach based on the adjusted Akaike information criterion, AICc (Symonds & Moussalli, 2010). We first identified the best-fitting (lowest AICc) model involving covariates only. Where several such models differed by less than 2 units of AICc, we retained them all as a set of best models. We then added developmental treatment and appropriate interactions to the best model or set of best models. Evidence for the importance of developmental treatment would take the form of reductions in AICc (that is, negative values of ΔAICc). Where multiple models are being considered, parameter estimates reported below are from the weighted average of all models under consideration, using R package ‘AICcmodavg’. The Appendix provides tables of AICc values for all the models discussed.

For physical comparisons of weight and wing length, the only candidate covariate was sex. For the flight performance variables, the basic set of candidate covariates was weight, wing length and (because of the possibility of habituation) escape number. To investigate trade-offs between different components of flight performance, we adopted the following strategy. First, we modelled take-off speed, using the basic set of covariates listed above. We then modelled take-off angle, using the basic set of covariates plus take-off speed (owing to the possible trade-off between take-off speed and take-off angle). Next, we modelled strings hit, using the basic set of covariates plus take-off speed and take-off angle (since manoeuvrability in the maze may have been affected by speed and angle of entry). Finally, we modelled time in flight, using the basic set of covariates plus all the other three flight performance measures. When considering developmental treatment, a main effect would demonstrate that developmental treatment altered the average level of that flight performance measure, whereas an interaction between developmental treatment and another component of flight performance would suggest that developmental treatment altered trade-offs between flight performance components.

### Ethical Note

Birds were taken from the wild under Natural England licence 20121066 and the research was completed under Home Office licence PPL60/4073, with approval of the local ethical review committee at Newcastle University. At the time of writing, the birds either are alive at Newcastle University or have been rehomed to outdoor aviaries.

The developmental manipulation involved cross-fostering. One chick of 48 that we cross-fostered in 2013 died between cross-fostering and the next morning; this is no greater than the expected rate of mortality this early in life. All other cross-fostered chicks gained weight between removal from their own nest and the next morning, suggesting rapid recovery from transport and acceptance in host nests. The manipulation may have increased developmental stress in the DIS group. However, the level of size discrepancy created is within the natural range observed in starling nests. Thus, the level of developmental stress is likely to have been within the naturally experienced range. Our manipulation was also as likely to improve a chick's position within its nest as to make it worse. The mean weights for the DIS group birds were not significantly lower than those of the ADV group at any weighing point; nor was the variance greater (Nettle et al., 2015). A total of two DIS birds and three ADV birds died before day 12; this is in line with rates of mortality in undisturbed nests in our starling colony.

The flight experiment is likely to have caused short-term stress due to social isolation, the unfamiliar environment and the startle stimulus. Birds were returned to their aviary within 72 h of being taken to the experimental arena. None showed any subsequent adverse effects. The maximum duration of the period during which birds were prevented from feeding by the experiment was 5.5 h.

## Results

The raw data are available as Supplementary Material. We successfully captured all flight performance measures for 76 escape flights from 20 birds (range 1–5 per bird, multiple flights from 18 birds). The six birds yielding no data consisted of five that did not come down to the near perch within the allotted time, and one for which we failed to capture video from all cameras. These six were drawn evenly from both developmental treatments (3:3), but all six were female. In addition to the escape flights, we recorded a routine flight from 11 birds. We report the analysis of the data from the escape flights only; including the routine flights does not alter any of the conclusions. There was considerable variation in how many minutes elapsed in the test sessions before the bird first came down to the near perch, but this variation was unrelated to developmental treatment (means + SD: ADV 140.67 + 167.43 min; DIS 157.78 + 163.17 min; adding developmental treatment to an intercept-only model predicting logged minutes to come down: ΔAICc = 2.97; B(DIS) = 0.25, 95% confidence interval, CI −1.30 to 1.79).

### Physical Measurements

Males were heavier than females on day 0 of the experiment (males 84.03 + 5.65 g, females 76.43 + 5.43 g). Adding sex to an intercept-only model predicting weight improved model fit (Table A1; B(Male) = 7.32, 95% CI 3.70–10.93). Further adding developmental treatment did not improve model fit (Table A1; B(DIS) = −0.51, 95% CI −3.90 to 2.88), and body weights were very similar between treatment groups (DIS 82.03 + 6.71 g, ADV 81.09 + 6.73 g). Likewise, males had longer wings than females (males 130.46 + 3.81 mm, females 125.21 + 5.30 mm; B(Male) = 3.76, 95% CI 1.14–6.38), but wing length was similar across treatment groups (DIS 129.50 + 4.42, ADV 128.12 + 5.39), and there was no support for adding developmental treatment to the model containing sex as a predictor of wing length (Table A2; B(DIS) = 0.65, 95% CI −1.81 to 3.10).

### Take-off Speed

**Figure 2:**
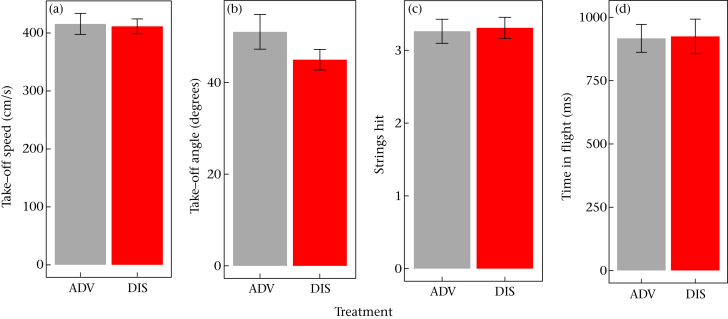
Between-bird means and SEs for each flight performance measure by developmental treatment. ADV: Advantaged birds; DIS: disadvantaged birds. (a) Take-off speed; (b) take-off angle; (c) number of strings hit; (d) time in flight.

We first modelled take-off speed. Four covariate-only models had AICc values within 2 units of one another (Table A3) and these were retained as a best set. Three of the four best models contained weight, and provide evidence overall for a negative effect of weight on take-off speed (*B* = −4.03, 95% CI −7.32 to −0.74). Adding developmental treatment to the models increased AICc in all cases (Table A3), and the parameter estimate for developmental treatment was close to zero (B(DIS) = −2.85, 95% CI −39.27 to 33.57). Thus, there was no support for take-off speed being affected by developmental treatment (between-bird mean + SD: ADV 415.42 + 60.04 cm/s, DIS 411.27 + 38.39 cm/s; Fig. 2).

### Take-off Angle

We next modelled take-off angle, including take-off speed as an additional predictor to account for possible trade-offs. Two covariates-only models were retained, the first containing weight and take-off speed and a lower-weighted model containing weight, take-off speed and wing length (Table A4). Across these two models, the effect of weight on take-off angle was negative (*B* = −1.02, 95% CI −1.74 to −0.30), and the effect of take-off speed was also negative (*B* = −0.06, 95% CI −0.11 to −0.01), suggesting a trade-off between take-off speed and take-off angle. Adding developmental treatment and its interaction with take-off speed to these models improved model fit in both cases (Table A4). DIS birds did tend to take off at slightly shallower angles (ADV 51.05 + 12.58 degrees, DIS 44.97 + 6.73 degrees; B(DIS) = −6.76, 95% CI −14.46 to 0.95; Fig. 2). However, the models containing both developmental treatment and its interaction with take-off speed fitted substantially better than those containing the main effect of developmental treatment alone (Table A4), suggesting that the effects of developmental treatment were operating via changes in the trade-off between take-off speed and take-off angle.

**Figure 3:**
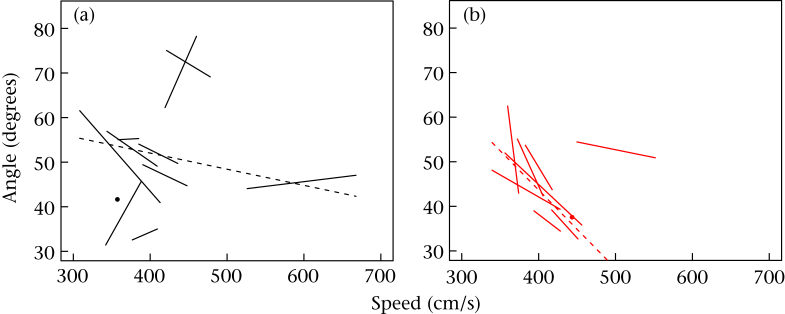
Representation of the trade-off between take-off angle and take-off speed for individual birds by developmental treatment. (a) Advantaged birds; (b) disadvantaged birds. Each solid line segment represents the fitted relationship from a regression of take-off angle on take-off speed for the escape flights of one bird, plotted from its lowest observed speed to its highest. Birds with a single recorded flight are shown as a point. The dashed line represents the central tendency for the group and uses the median intercept and median slope.

To explore the interaction further, for each bird for whom we had more than one escape flight, we fitted a linear regression model of take-off angle on take-off speed (see Fig. 3). For the DIS birds, these slopes were uniformly negative (median −0.18). For the ADV birds, the slopes were not so consistently or strongly negative (median −0.04).

### Strings Hit

For strings hit, a total of four models were retained in the best set of covariate-only models (Table A5). However, the best-supported of these was the intercept-only model, and the other three had no parameters in common. Thus, there was no strong support for any of the covariates contributing substantially to explaining variation in the number of strings hit. The mean number of strings hit was similar across the two developmental treatments (ADV 3.26 + 0.55, DIS 3.31 + 0.43; Fig. 2), and adding developmental treatment to the models increased AICc in every case (Table A5; B(DIS) = 0.03, 95% −0.44 to 0.49).

### Time of Flight

For time of flight, five best-fitting covariate-only models were retained (Table A6). All five contained take-off angle and take-off speed, and four of the five, including the two most heavily weighted, contained number of strings hit. The parameter estimate for take-off speed was negative, meaning flights with faster take-offs took less time overall (*B* = −1.27, 95% CI −2.02 to −0.53). The effect of take-off angle was positive: steeper take-off angles made flights longer (*B* = 8.83, 95% CI 5.77–11.89). This was due to the steeper angles increasing the distance travelled. The most direct path from perch to perch involved a take-off angle of approximately 31°, and the steeper angles involved the birds reaching the ceiling well short of the far perch and having to change direction. The effect of strings hit tended to be positive, suggesting birds took longer when they had hit more strings (*B* = 24.75, 95% CI −5.46 to 54.95). Adding developmental treatment and its interaction with take-off angle to the models slightly improved fit in all cases (Table A6). Models containing the developmental treatment by take-off angle interaction were in all cases superior to those containing the main effect of developmental treatment alone (Table A6). There was no support for models containing any additional interactions (data not shown).

**Figure 4:**
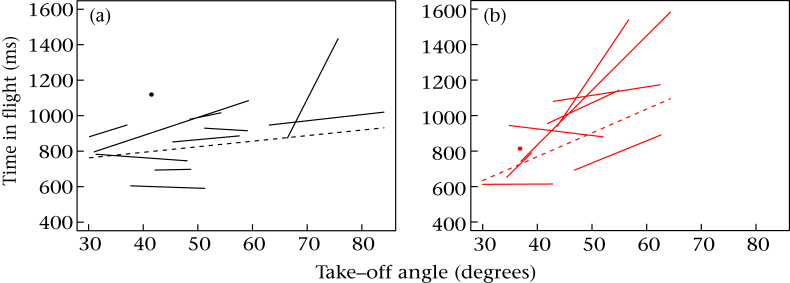
Representation of the trade-off between take-off angle and total time in flight for individual birds by developmental treatment. (a) Advantaged birds; (b) disadvantaged birds. Each solid line segment represents the fitted relationship from a regression of time in flight on take-off angle for one bird, plotted from its lowest observed angle to its highest. Birds with a single recorded flight are shown as a point. The dashed line represents the central tendency for the group and uses the median intercept and median slope.

To visualize the interaction between developmental treatment and take-off angle, we again calculated slopes of time of flight against take-off angle for individual birds. The slopes for the DIS birds were more strongly positive (median 0.02) than those for the ADV birds (median 0; Fig. 4). Because of this, ADV birds were able to maintain very similar times of flight to DIS birds (ADV 1.15 + 0.23 s, DIS 1.16 + 0.26 s; Fig. 2), despite tending to take off at steeper angles.

## Discussion

By measuring multiple components of flight performance separately, and retaining information from multiple flights from the same bird, we were able to confirm the presence of trade-offs in flight performance. For a bird to increase the steepness of its take-off angle, it had to reduce its take-off speed, in line with previous findings from both starlings and other passerines (Kullberg et al., 1996; Lind et al., 2010; Witter et al., 1994). Increasing the steepness of the take-off angle also increased the total time in flight. The reason for this was that the total distance travelled became greater, with birds setting off at a steep angle reaching the ceiling of the arena well before the far perch, and having to change direction to fly horizontally along under the ceiling. The only trade-off of which we found no evidence was that between speed and accuracy of manoeuvre in the aerial maze (Brilot et al., 2009); neither faster take-off speed nor shorter overall time of flight was associated with more strings hit.

The effects of developmental treatment on mean flight performance were not marked. Of the four flight performance measures, only one, take-off angle, gave any indication of differing between the two developmental treatment groups, with developmentally disadvantaged birds tending to take off at shallower angles than their advantaged siblings. However, closer analysis revealed that developmental stress intensified the trade-offs that birds faced between different components of flight performance. Birds from the disadvantaged treatment had on average to sacrifice more take-off speed for every degree of take-off angle gained, and likewise suffered a greater decrement in time to reach their destination for every additional degree of take-off angle, than the advantaged individuals. Thus, advantaged birds were able to reach their destination just as quickly as disadvantaged birds, despite having taken off at steeper angles. The disadvantaged birds appeared to sacrifice take-off angle in order to defend take-off speed, a pattern that has also been observed with artificially weighted starlings (Witter et al., 1994).

Birds from the two developmental treatments did not differ significantly in weight or wing length at the time of testing. Thus, it seems likely that the developmental manipulation had caused subtle physiological differences that were still persistent in the adult birds. Although we did not measure stress hormones directly during our developmental manipulation, the accelerated telomere attrition shown by the disadvantaged birds suggests they experienced greater levels of physiological stress (Nettle et al., 2015). Stress hormone exposure during development affects muscle structure and function in a number of ways (Dong et al., 2007). It is possible that increased stress caused by our manipulation constrained the development of muscular capacity, meaning that the disadvantaged birds were not able to produce the muscular force to mitigate the negative effects of taking off steeply on speed to the same extent as the advantaged birds. Alternatively, developmental stress may have increased the physiological cost of flying with a given velocity, leading disadvantaged birds to economize on steepness. It has been shown in budgerigars, *Melopsittacus undulatus*, that high-quality diet in adulthood reduces the cost of flight in terms of oxidative damage (Larcombe et al., 2008), and benign developmental circumstances could have a similar effect.

Our results are in accord with previous studies that have found ‘silver spoon’ effects: lasting negative effects of posthatching developmental stress on adult flight performance in birds (Criscuolo et al., 2011; Miller, 2011; Verspoor et al., 2007). We found no evidence for any adaptive improvement of flight performance by developmental stress, as found by two studies that manipulated prehatching stress exposure (Chin et al., 2009; Coslovsky & Richner, 2011). Thus, our study fits into the general pattern that posthatching developmental stress has negative effects on flight performance, whereas prehatching developmental stress may instead induce adaptive plasticity.

The observed pattern of trade-offs between different components of flight performance, and the effects of developmental history on the strength of those trade-offs, illustrates the importance of analysing data from multiple flights from the same individuals, rather than taking a single flight or a mean level of flight performance per bird. It also illustrates the value of analysing each component of flight performance separately for many purposes, rather than reducing flight performance to a single index. The only one of our measures not to show any interpretable relationship to our predictors was the number of strings hit in the aerial maze. This measure proved difficult to score accurately from the videos, and our inter-rater agreement was poor. Thus, the lack of pattern may reflect imprecision of our measurement. Although we have successfully used a similar method before (Brilot et al., 2009), more reliable methods for assessing manoeuvrability might be provided by using solid obstacles and marking birds' wings with ink (Swaddle et al., 1996).

There is widespread evidence from birds that developmental stress can have a negative impact on survival and on fitness (Lindström, 1999). These effects must be mediated through some aspects of the individual's performance or capacity. The present results are striking since the developmental manipulation was so subtle, altering only the slight initial weight differences between the focal chicks and their competitors, and yet its influence was detectable in adulthood. The results demonstrate that small developmental stressors can have lasting physiological impacts even without obviously affecting growth patterns or adult size. Subtle differences in the circumstances of early development could thus have far-reaching implications for adult fitness and behaviour; there may be both direct fitness impacts due to reduced performance and compensatory behavioural changes such as risk avoidance and increased vigilance as a response of the individual to its own state (Lind & Cresswell, 2005).

## Figures and Tables

**Table A1:** Models for weight on day 0 of the experiment

Model	*k*	AIC_c_	Δ AIC_c_
Intercept only	3	142.01	0.00
Sex	4	132.20	−9.81^a^
Sex+Treatment	5	135.60	3.39^b^

a Relative to intercept-only model.

b Relative to model with sex only.

**Table A2:** Models for wing length

Model	*k*	AIC_c_	Δ AIC_c_
Intercept only	3	125.20	0.00
Sex	4	120.33	−4.86^a^
Sex+Treatment	5	123.50	3.17^b^

a Relative to intercept-only model.

b Relative to model with sex only.

**Table A3:** Models for take-off speed

Model	*k*	AIC_c_	Δ AIC_c_	Weight
**Best covariate-only models**				
Intercept only	4	785.87	0.00^a^	0.36
Weight	5	786.13	0.26^a^	0.31
Weight+Escape number	6	787.27	1.40^a^	0.18
Weight+Wing length	6	787.58	1.71^a^	0.15
**Models with developmental treatment**				
Treatment	5	788.14	2.27^b^	0.36
Weight+Treatment	6	788.42	2.29^b^	0.31
Weight+Escape number+Treatment	7	789.61	2.34^b^	0.17
Weight+Wing length+Treatment	7	789.83	2.25^b^	0.15

a Relative to best model, which is intercept-only.

b Relative to corresponding covariate-only model.

**Table A4:** Models for take-off angle

Model	*k*	AIC_c_	Δ AIC_c_	Weight
Intercept only	4	561.09	–	–
**Best covariate-only models**				
Speed+Weight	6	556.41	0.00^a^	0.72
Speed+Weight+Wing Length	7	558.35	1.94^a^	0.28
**Models with developmental treatment and interaction**				
Speed+Weight+Treatment+Treatment*Speed	8	553.11	−3.30^b^	0.70
Speed+Weight+Wing Length+Treatment+Treatment*Speed	9	554.76	−3.59^b^	0.30
**Models with developmental treatment main effect only**				
Speed+Weight+Treatment	7	556.24	3.13^c^	0.69
Speed+Weight+Wing length+Treatment	8	557.83	3.07^c^	0.31

a Relative to best covariate-only model.

b Relative to corresponding covariate-only model.

c Relative to corresponding model with main effect and interaction.

**Table A5:** Models for strings hit

Model	*k*	AIC_c_	Δ AIC_c_	Weight
**Best covariate-only models**				
Intercept only	4	227.89	0.00^a^	0.36
Weight	5	228.69	0.81^a^	0.24
Escape number	5	228.79	0.90^a^	0.23
Wing length	5	229.40	1.51^a^	0.17
**Models with developmental treatment**				
Treatment	5	230.16	2.27^b^	0.37
Weight+Treatment	6	231.04	3.15^b^	0.24
Escape number+Treatment	6	231.15	3.26^b^	0.23
Wing length+Treatment	6	231.75	3.86^b^	0.17

a Relative to best model, which is intercept-only.

b Relative to corresponding covariate-only model.

**Table A6:** Models for time in flight

Model	*k*	AIC_c_	Δ AIC_c_	Weight
Intercept only	4	1020.79	–	–
**Best covariate-only models**				
Angle+Speed+Hits+Escape number	8	985.58	0.00^a^	0.28
Angle+Speed+Hits	7	985.60	0.02^a^	0.28
Angle+Speed	6	986.13	0.55^a^	0.21
Angle+Speed+Hits+Weight	8	987.16	1.58^a^	0.13
Angle+Speed+Hits+Escape number+Weight	9	987.48	1.90^a^	0.11
**Models with developmental treatment and interaction**				
Angle+Speed+Hits+Escape number+Treatment+Treatment*Angle	10	983.89	−1.69^b^	0.33
Angle+Speed+Hits+Treatment+Treatment*Angle	9	984.45	−1.15^b^	0.25
Angle+Speed+Treatment+Treatment*Angle	8	985.12	−1.01^b^	0.18
Angle+Speed+Hits+Escape number+Weight+Treatment+Treatment*Angle	11	986.00	−1.48^b^	0.12
Angle+Speed+Hits+Weight+Treatment+Treatment*Angle	10	986.04	−1.44^b^	0.11
**Models with developmental treatment main effect only**				
Angle+Speed+Hits+Treatment	8	985.73	1.84^c^	0.27
Angle+Speed+Hits+Escape number+Treatment	9	986.00	2.11^c^	0.23
Angle+Speed+Hits+Weight+Treatment	9	986.40	0.36^c^	0.19
Angle+Speed+Treatment	7	986.47	1.35^c^	0.18
Angle+Speed+Hits+Escape number+Weight+Treatment	10	987.16	1.16^c^	0.13

a Relative to best covariate-only model.

b Relative to corresponding covariate-only model.

c Relative to corresponding model with main effect and interaction.
